# What are those cilia doing in the neural tube?

**DOI:** 10.1186/2046-2530-1-19

**Published:** 2012-10-01

**Authors:** Sarah N Bay, Tamara Caspary

**Affiliations:** 1 Department of Human Genetics, Emory University School of Medicine, Atlanta, GA 30322, USA; 2 Graduate Program in Genetics and Molecular Biology, Atlanta, GA, 30322, USA

**Keywords:** Cilia, neural tube development, PCP signaling, Shh signaling

## Abstract

Primary cilia are present on almost all vertebrate cells, and they have diverse functions in distinct tissues. Cilia are important for sensation in multiple capacities in contexts as different as the retina, kidney, and inner ear. In addition to these roles, cilia play a critical part in various developmental processes. Of particular importance is the development of the neural tube, where cilia are essential for the transduction of the Sonic Hedgehog (Shh) signaling pathway that specifies neuronal cell fates. This relationship is well established and is the most recognizable function for cilia in the neural tube, but it may be part of a larger picture. Here, we discuss the links between cilia and Shh signaling, as well as suggesting additional roles for cilia, and mechanisms for their placement, in the neural tube.

## Review

Since being functionally linked to the Sonic Hedgehog (Shh) signaling pathway, primary cilia have sparked enormous interest. The initial connection came from an unbiased forward genetic mouse screen in which a number of the mutations disrupted genes important for cilia; the resulting mutant embryos showed abnormal patterning of the neural tube [1,2]. Shh signaling controls neural tube patterning [3,4], and double mutant analysis showed cilia are critical for Shh signal transduction [1,2]. Previously, the focus of most research was on the function of motile cilia, which have a 9 + 2 microtubule structure, but the past decade has witnessed an explosion of interest in primary cilia, which lack the inner doublet and instead have a 9 + 0 axonemal arrangement. These cilia have now been implicated in many biological processes, from obesity to cancer to learning and memory [5-8]. Indeed, the widespread role of cilia in various systems is made clear by the range of phenotypes present in the ciliopathies, which are human diseases that arise from mutations in cilia genes [9-13]. In this review, we return to the beginning and the source of all the excitement - the embryonic neural tube.

Core progress has been made towards understanding the mechanistic details behind the abnormal neural patterning of mouse mutants with disrupted cilia [1,2,14,15], but other roles for cilia in the neural tube have yet to be explored. Proper positioning of cilia in several developmental contexts is linked to the planar cell polarity (PCP) pathway, raising the possibility that the placement of cilia in the neural tube may be critical [16-21]. Additionally, specialized ependymal cilia control circulation and mechanosensation of cerebral spinal fluid (CSF) in the ventricles of the brain [22-25], which is derived from the anterior neural tube. Taken together, these data give us a glimpse of what the cilia in the neural tube are really doing there.

### Formation of the neural tube

Following the specification of the germ layers, the neural tube starts to form [26,27]. The process proceeds in three dimensions, most critically along the dorsal-ventral (D-V) and anterior-posterior (A-P) body axes. Within the D-V axis, the dorsal ectoderm thickens into the neural plate (or neuroepithelium) whose borders then elevate into neural folds and subsequently extend to roll into a tube. The tops of the neural folds fuse along the midline to close the neural tube, and the structure then separates from the overlying surface ectoderm. The floor plate at the ventral midline is induced by Shh from the underlying notochord, and neural patterning proceeds as cell fates are specified along the D-V axis. At the same time, along the A-P axis, the tube narrows and elongates in a process termed convergent extension (CE); errors in this step lead to neural tube defects (NTDs), such as craniorachischisis, anencephaly, and spina bifida [28].

### Cilia are required for Sonic Hedgehog signaling and neural tube patterning

Patterning of the neural tube and correct cell fate specification are integral parts of proper development, and tight control of the Shh signaling pathway is required for appropriate ventral patterning and specification of motor neurons and interneurons [29,30]. The dorsal cell fates are specified by the BMP and Wnt signaling pathways. As the links between cilia and Wnt signaling are controversial, they are well reviewed elsewhere [20,31-36]. Clearly, however, cilia are known to be required for Shh signaling in mammals, with many members of the pathway localizing to cilia [37-39].

In the ventral neural tube, a combination of the amount and duration of Shh signaling specifies six neural progenitor cell fates [29,30,40]. Due to their proximity to the underlying notochord (the initial source of secreted Shh), the most ventral cells at the midline are exposed to the highest concentrations of Shh and are specified as the floor plate and the p3 domain, which will give rise to V3 interneurons [29,35,41,42]. Intermediate levels of Shh induce the formation of the pMN, p2, p1, and p0 domains, precursors to motor neurons and V2, V1, and V0 interneurons, respectively [29,35]. The presence of Shh also inhibits dorsal cell type specification [40]. One of the key features of the Shh gradient that directs patterning is the balance between activator and repressor transcriptional activities. As cells are exposed to varying levels of Shh, a signaling cascade is responsible for the regulation of the Gli family of transcription factors (Gli1 to Gli3), which act as effectors that can activate (GliA) or repress (GliR) transcription of target genes. Gli1 acts solely as an activator, and, though Gli2 and Gli3 both contain repressor domains, Gli2 acts as the primary activator while Gli3 is the major repressor in the neural tube [43-46]. It is ultimately the balance of these two opposing signals that is critical for patterning, and cilia are crucial for maintaining this balance.

The transduction of the signal from Shh ligand to the Gli transcription factors is an intricate cascade involving many elements, including Patched (Ptch1), a transmembrane receptor, Smoothened (Smo), a membrane protein, Suppressor of Fused (SuFu), and Kif7, a possible motor protein. In the absence of Shh, Ptch1 inhibits Smo and causes repression of the pathway [39]. This results in the proteolytic cleavage of Gli3, the major repressor, and the subsequent repression of target genes [46]. When Shh ligand is present, it binds to Ptch1, which removes the repression of Smo [39]. Gli3 is no longer cleaved into the repressor form, Gli2 is stabilized and activated, and the transcription of target genes, among them *Ptch1* and *Gli1*, is promoted.

In vertebrate systems, this signaling cascade happens in the context of the cilium, and the dynamic transport of pathway members is key for proper transduction. When the pathway is off, Ptch1 is localized to the cilium whereas Smo is not; upon stimulation by Shh ligand, Ptch1 leaves the cilium while Smo becomes enriched there [39]. The Gli proteins, Kif7, and SuFu all localize to the tips of cilia [47-50]. Gli proteins are bound and stabilized by SuFu and bidirectionally trafficked along the axoneme; without pathway activation, they are cleaved to produce GliR [2,49,50]. SuFu also acts to inhibit the activation of Gli [49,50]. Kif7, the mouse homolog of *Drosophila* Costal-2, functions between Smo and the Gli proteins to both negatively and positively regulate the pathway [47]. Though they are not the only regulators, core cilia proteins function prominently in pathway control.

### Intraflagellar transport mutants connect cilia transport and Sonic Hedgehog signaling

Cilia and Shh were initially linked through forward genetics in the mouse, since mutants defective in intraflagellar transport (IFT) proteins showed improper neural tube patterning, and IFT is necessary for ciliogenesis and cilia maintenance [1,2]. The IFT particles are composed of two biochemically distinct complexes: IFTA and IFTB [51]. IFTB proteins, along with the molecular motor kinesin-II, are necessary for transport from the base of the cilium to the tip (anterograde transport), and it was mutations in members of this complex that gave the first indication that cilia and Shh signaling were connected. Mutations in IFTB genes, such as *Ift88* and *Ift172*, lead to either absent or severely shortened cilia; this results in no Shh signaling and a lack of both GliA and GliR. Thus, in *Ift88*^*pol*^ and *Ift172*^*wim*^ mutants, the lack of activation combined with derepression causes neural tube mispatterning [1,2].

Together, IFTA proteins and the cytoplasmic motor dynein power retrograde transport, which traffics proteins from the tip of the cilium back toward the cell [51]. Unlike anterograde transport mutants, which lack cilia, retrograde transport mutants have abnormal cilia morphology due to protein accumulation at the tip of the cilium [48]. This can result in complete blockage of signal transduction (as in Dync2h1, discussed below) or in ectopic pathway activation, as seen in the *Ift122* null allele *sister of open brain (sob)*[48,52]. These retrograde defects highlight the fact that the movement of Gli proteins into the cilium is not sufficient to support their proper activity. IFT proteins are not only crucial to build and maintain the cilium, but also to regulate Gli activator and repressor function [48,52].

Though originally a topic of intense debate, the claim that transduction of the Shh pathway is intricately tied to proper cilia structure and transport has been supported by the identification of diverse mutants that all disrupt cilia structure or impair cilia protein transport. The simple ability to move pathway components into and out of the cilium is not enough for proper signal transduction; instead, the relative rates of anterograde and retrograde transport are critical. This is demonstrated most strikingly by the genetic interactions of anterograde and retrograde trafficking mutants. Dync2h1 is the heavy chain of dynein, the cytoplasmic motor responsible for retrograde transport, and when it is disrupted (as in its mutant *ling-ling*), Ptch1, Smo, and Gli2 are all trafficked into the cilium, but no Shh transduction occurs, causing a dorsalized neural tube [52,53]. Interestingly, a single copy of a hypomorphic allele of the anterograde IFTB component, Ift172 (*Ift172*^*avc1*/*+*^), rescues the patterning phenotype caused by the absence of retrograde transport [52]. Even more intriguing is the demonstration that reduction of IFT122, an IFTA protein, via *Ift122*^*sopb*/+^ is also able to suppress the *Dync2h1*^*lln*/*lln*^ phenotype [52]. Though both Dync2h1 and Ift122 are assumed to be part of the retrograde transport complex, the fact that *Ift122*^*sopb*/+^ rescues the *Dync2h1*^*lln*/*lln*^ phenotype suggests that Ift122 may also act outside retrograde transport, emphasizing the nuances of transport necessary for proper signaling. This is further highlighted by recent analysis of an allelic series of IFTA mutants, which demonstrated that proper Shh signaling relies on correct cilia architecture and protein trafficking [54].

In addition to the conserved cilia proteins critical in building and maintaining cilia through multiple phyla, other genes that are important for cilia also regulate Shh signaling. For example, Arl13b and Rab23 are small GTPases, and mutations in either lead to unique defects in neural tube patterning. Strikingly, this is due to loss of Arl13b preventing full activation of GliA but leaving normal GliR function intact [55]. In genetic analysis, Arl13b functions downstream of Smo, but cell biological analysis shows that it also controls entry of Smo into the cilium, arguing that it may possess an additional upstream function [55,56]. Similarly, Rab23 may function to affect cilia trafficking at multiple points. Rab23 mutants display ventralized neural tube patterning, and genetic analysis has placed it downstream of Smo, suggesting that it functions primarily through inhibiting the activation of Gli2 [57-59]. More recently, quantitative analysis of protein trafficking in the cilium has described a role for Rab23 in overseeing the recycling rate of Smo [60]. Furthermore, loss of another Shh inhibitor, TULP3, leads to ventralization of the neural tube [61,62]. TULP3, a tubby-like protein, acts to repress Shh in the absence of ligand in a Gli2-dependent but Smo-independent manner and is vital to balancing progenitor proliferation with neuron differentiation [61,62]. Pathway regulators such as these, which act at multiple steps of the cascade, suggest that the cilium as an organelle may function for efficiency; this is to say, its small and controlled environment appears to have evolved to use some proteins in many different capacities as a way to maximize the effectiveness of the system.

### Right place … right time

Although the intricate connections between cilia and Shh signal transduction are the best understood at present, the cilia within the neural tube may have additional functions. Cilia on cells that line the ventricular zone of the neural tube are aligned and extend into the lumen. This organization is most obvious at the most ventral levels of the ventricular zone near the floor plate. It seems unlikely that this specific orientation is an artifact but rather suggests that the placement of these cilia may be important to their function, to their ability to respond to signal, or to the morphology of the neural tube. For example, it is not yet known whether cilia in the neural tube function solely to transduce the Shh or if they actually sense the Shh ligand. Perhaps this organization of cilia in the ventral neural tube is most obvious as their length there is proportional to their ability to detect Shh ligand. On the other hand, the position of the cilium directly relates to the plane of cell division. As the cells lining the lumen are highly proliferative progenitor cells, a subset of which remain progenitors, the positioning of the cilium could be critical for cellular asymmetry. Shh has a well established role as a mitogen in other tissues [63,64], and the cells in the ventricular zone are highly proliferating progenitors; the cilia could be present for morphogenesis in addition to patterning. Cilia position is critical to proper function in other developmental contexts. For example, PCP, or non-canonical Wnt signaling, controls cilia localization in the embryonic node [19]. Should cilia position in the neural tube be shown to be deliberate, PCP signaling is an excellent candidate for overseeing this process. Additionally, cilia are known for their sensory function in other contexts. In ependymal cells, which line the ventricles of the brain and are responsible for CSF production and circulation, cilia are known to have mechano- and chemosensory roles [23,25]. Thus, it is reasonable to speculate that properly oriented cilia in the neural tube may sense multiple cues.

### Planar cell polarity signaling is responsible for cilia orientation

CE is critical to neural tube formation as it elongates the tissue so that the brain and spinal cord eventually form. Cells that participate in CE must be polarized, and this specific cell shape is dependent on the PCP cascade [17,28,65,66]. While this role for PCP in the neural tube is well established, recent research connecting PCP signaling to cilia positioning points to another way this pathway could function in the neural tube. If PCP signaling influences cilia orientation in the neural tube, as it does in other developmental contexts, this would provide a mechanism for the cilia to be in the right place at the right time.

Analyses of mutants in either PCP signaling or ciliogenesis indicate that neither PCP signaling nor CE require cilia. Mutants with disrupted cilia display normal CE, and cilia play no known role in the process. Despite this, a variety of data link PCP signaling and cilia [16-20], raising the possibility that there may be interplay between the two in the neural tube. Mouse models of the human ciliopathy Bardet-Biedl syndrome (BBS) disrupt any of 12 BBS genes, which are localized to the basal body and cilium and have phenotypes reminiscent of PCP mutants [16]. Mutations in either *Inturned* or *Fuzzy*, which regulate PCP signaling and CE, also display abnormal ciliogenesis and secondary Shh defects [17,21]. Together, these data indicate that PCP signaling and cilia have a relationship. It appears that PCP function is necessary for cilia orientation, which then underlies cilia function. In the embryonic node, PCP signaling positions cilia in order for the flow necessary for left-right axis specification to be established [19]. Furthermore, a core PCP component, Vangl2, genetically interacts with the core cilia machinery protein, IFT88, in kinocilia in the organ of Corti [18]. Together, these data show the reliance of cilia function on proper placement and orientation of the cilium.

Given the role of PCP in cilia orientation, it is vital to determine whether or not PCP signaling influences the position and/or organization of cilia in the ventricular zone of the neural tube. The first step would be to examine cilia in the neural tube of known PCP mutants to determine if the anecdotal arrangement of cilia is disturbed. Finding a way to disrupt the placement of cilia will confirm that their position is indeed deliberate, and from there we will be able to pose the question of mechanism and function.

### A sensory role

What remains unclear is whether the cilia in the neural tube are there solely to transduce Shh signaling. After all, in the lungs, motile cilia that clear mucus also express taste receptors [67], so perhaps all cilia can perform multiple functions. Several systems are suggestive. For instance, the *Caenorhabditis elegans* nervous system is ciliated, and those cilia are critical for chemosensation [68]. In the mammalian brain, sensory cilia are found on ependymal cells, which are multiciliated epithelial cells that line the ventricles of the brain [23,25]. Additionally, these cilia are motile and beat to circulate CSF. Mature ependymal cells develop from radial glial precursors, whose cilia regulate polarization of the mature ependymal cells, underscoring the importance of cilia orientation [22-24]. Furthermore, cells in the choroid plexus possess clusters of primary cilia responsible for CSF production, and loss of these cilia causes defects in autocrine signaling and transcytosis, leading to misregulation of CSF production [25]. These data establish a sensory role for many diverse cilia and make us wonder whether cilia of the neural tube could also have such a function. Additionally, the idea that neural tube cilia may have a sensory function similar to that of the motile cilia discussed raises the question of whether motile cilia could also be playing a role in Shh signaling. Could both sensory and signaling functions be common to multiple types of cilia?

## Conclusion

Since a mouse mutant linked Shh and cilia almost a decade ago, the field has focused on understanding the mechanistic links between the two. This work has painted a dynamic picture, whereby key components move into and out of cilia to regulate the ratio of GliA and GliR and ultimately specify distinct cell fates in the neural tube. Other signaling pathways have also been linked to cilia, although whether such links are critical in the neural tube remains unclear. The projection of cilia into the lumen of the ventricular zone strongly suggests that they could play a sensory role, in which case the positioning of these cilia would be crucial and highly regulated, possibly through PCP signaling. But the biggest question remains: do these cilia play another functional role? Cilia in other contexts are strikingly suggestive, and the field is poised to investigate for what other tasks, if any, the cilia of the neural tube may be responsible.

## Abbreviations

A-P: anterior-posterior; BBS: Bardet-Biedl syndrome; CE: convergent extension; CSF: cerebral spinal fluid; D-V: dorsal-ventral; IFT: intraflagellar transport; NTD: neural tube defect; PCP: planar cell polarity; Ptch1: Patched; Sh: Sonic Hedgehog; Smo: Smoothened; SuFu: Suppressor of Fused.

## Competing interests

The authors declare that they have no competing interests.

## Authors’ contributions

SNB and TC wrote the manuscript. Neither has any conflict of interest to report. Both authors read and approved the final manuscript.
